# Parents’ understanding of genome and exome sequencing for pediatric health conditions: a systematic review

**DOI:** 10.1038/s41431-022-01170-2

**Published:** 2022-08-23

**Authors:** Jessica Gereis, Kate Hetherington, Lauren Ha, Eden G. Robertson, David S. Ziegler, Kristine Barlow-Stewart, Katherine M. Tucker, Jonathan M. Marron, Claire E. Wakefield

**Affiliations:** 1 School of Clinical Medicine, UNSW Medicine & Health, UNSW Sydney, Sydney, NSW Australia; 2 Behavioural Sciences Unit, Kids Cancer Centre, Sydney Children’s Hospital, Sydney, NSW Australia; 3 School of Health Sciences, UNSW Medicine & Health, UNSW Sydney, Sydney, NSW Australia; 4 Kids Cancer Centre, Sydney Children’s Hospital, Sydney, NSW Australia; 5 Children’s Cancer Institute, UNSW Sydney, Sydney, NSW Australia; 6 Northern Clinical School, Faculty of Medicine and Health, University of Sydney, Sydney, NSW Australia; 7 Prince of Wales Clinical School, UNSW Medicine & Health, UNSW Sydney, Sydney, NSW Australia; 8 Department of Pediatric Oncology, Dana-Farber Cancer Institute, Boston, MA USA; 9 Division of Hematology/Oncology, Boston Children’s Hospital, Boston, MA USA; 10 Center for Bioethics, Harvard Medical School, Boston, MA USA

**Keywords:** Patient education, Paediatrics

## Abstract

Genome and exome sequencing (GS/ES) are increasingly being used in pediatric contexts. We summarize evidence regarding the actual and perceived understanding of GS/ES of parents of a child offered testing for diagnosis and/or management of a symptomatic health condition. We searched four databases (2008–2021) and identified 1264 unique articles, of which 16 met inclusion criteria. We synthesized data from qualitative and quantitative studies and organized results using Ayuso et al. (2013)’s framework of key elements of information for informed consent to GS/ES. Many of the parents represented had prior experience with genetic testing and accessed a form of genetic counseling. Parents’ understanding was varied across the domains evaluated. Parents demonstrated understanding of the various potential direct clinical benefits to their child undergoing GS/ES, including in relation to other genetic tests. We found parents had mixed understanding of the nature of potential secondary findings, and of issues related to data privacy, confidentiality, and usage of sequencing results beyond their child’s clinical care. Genetic counseling consultations improved understanding. Our synthesis indicates that ES/GS can be challenging for families to understand and underscores the importance of equipping healthcare professionals to explore parents’ understanding of ES/GS and the implications of testing for their child.

## Introduction

Identification of clinically actionable pathogenic or likely pathogenic gene variants through comprehensive genome and exome sequencing and analysis (GS/ES) has enabled advances in the diagnosis and treatment of pediatric health conditions such as congenital anomalies and childhood cancer [1]. While these advances bring considerable potential benefits, the application of GS/ES is complex. Psychosocial and ethical issues that may arise from GS/ES are related to achieving informed consent, privacy and confidentiality, the limitations of testing, and the discovery of variants of uncertain significance or secondary findings [1–3]. Ayuso et al. have developed a framework of key elements of information to be addressed during informed consent processes for GS/ES [4].

Understanding of GS/ES constitutes a primary challenge for genetic counselors and related healthcare professionals in facilitating informed consent processes for GS/ES [3]. Caregivers of pediatric patients (herein called “parents”) can often overestimate the potential “promise” of GS/ES-based technologies [5, 6]. Low levels of health and genomic literacy can be barriers to parents achieving strong understanding of GS/ES and related concepts [3, 7]. The emotional challenge often following news of a child’s serious health condition can further impede comprehension of complex information [8]. Where GS/ES is offered as part of a research study alongside clinical care, there is an increased risk of subjects conflating the understanding acquisition goals of research with the therapeutic goals of clinical care [5, 6, 9].

Important goals of informed consent processes include that individuals be both well informed according to rigorously-defined metrics and perceive themselves to be well informed via self-report [10]. In this study, we delineate understanding into “actual” and “perceived” understanding. We define “actual understanding” as an individual’s verifiable understanding whereby accuracy and depth of understanding can be determined, and, “perceived understanding” referring to an individual’s degree of belief that they are well informed [10]. Perceived understanding plays a role in problem-solving and decisional involvement, and consistency between an individual’s perceived and actual understanding are expected to be critical for facilitating the decision-making process and driving individuals’ information seeking behaviors [11, 12]. Identification of gaps in parents’ actual and perceived understanding of GS/ES can highlight areas for greater focus in consultations with families and inform development of information resources. This systematic review therefore aimed to summarize available evidence of:

1. Parents’ *actual* understanding of GS/ES-related concepts,
2. Parents’ *perceived* understanding of GS/ES-related concepts, and
3. Factors associated with parents’ actual and/or perceived understanding of GS/ES-related concepts

## Materials and methods

### Search strategy

We conducted a systematic search of peer-reviewed literature across four databases (Ovid MEDLINE, Embase, APA PsycINFO, and CINAHL), adhering to PRISMA guidelines [13]. We also searched Google Scholar, articles citing included articles, and the reference lists of included articles. JG, KH, CEW, DSZ, and EGR devised the search strategy by combining keywords relevant to GS/ES in pediatric settings and parent understanding (Supplementary Methods). We used EndNote X9 to collate abstracts and perform deduplication. Using Rayyan [14], an online article management tool designed for systematic reviews, JG and LH independently screened all unique abstracts and achieved consensus on disagreements through discussion, and calculated the Cohen’s kappa to determine interrater reliability [15]. Using Excel spreadsheets, JG and LH screened the full texts of the remaining articles, again achieving consensus through discussion.

We included studies published in English from January 2008 to March 2021. We selected 2008 as the start of our search period due to the significant GS/ES-related advances that coincide with this date [16]. We included peer-reviewed articles that evaluated actual and/or perceived understanding of GS/ES in parents of a child with a symptomatic health condition that led to them being offered GS/ES, including studies conducted in research, clinical or hybrid contexts. Due to practical and ethical differences between testing contexts, we have narrowed the scope for included publications to those focusing on populations where testing was conducted in response to a child having a clinically presenting health condition, and excluded articles where testing was indicated for pre-symptomatic adult-onset conditions, or was conducted prenatally or for reproductive decisions.

### Article quality assessment

We assessed eligible studies for quality and risk of bias using the Mixed Methods Appraisal Tool (MMAT) (Supplementary Table 2) [17]. One author (JG) assessed the quality of all included studies, and a second author (LH) independently performed quality assessment of a subset to ensure reliability.

### Data extraction

We extracted study-specific information from articles including study design, study objectives, measures used for evaluating understanding, type of GS/ES conducted, details of the context in which GS/ES and the study was performed, sociodemographic information of the sample, and stage in the “clinical journey” when understanding was assessed (i.e., before or after the return of GS/ES results). We also summarize key findings relevant to our aims. Where it was reported, we summarized relevant details about genetic counseling and information provided to families prior to consenting to GS/ES for their child. The first author (JG) extracted the data from all included studies, and a second author (LH) independently extracted data for a subset of the included studies (randomly selected) to ensure accuracy.

### Analysis

The first author (JG) coded extracted data relevant to the review aims from all studies and synthesized findings on the domains of understanding. Heterogeneity of study methodologies and outcomes precluded meta-analysis of the included studies. Where possible, we organized results using Ayuso et al. [4] framework of key elements of information for informed consent to participation in genomic research (described in Supplementary Table 1).

## Results

**Fig. 1:**
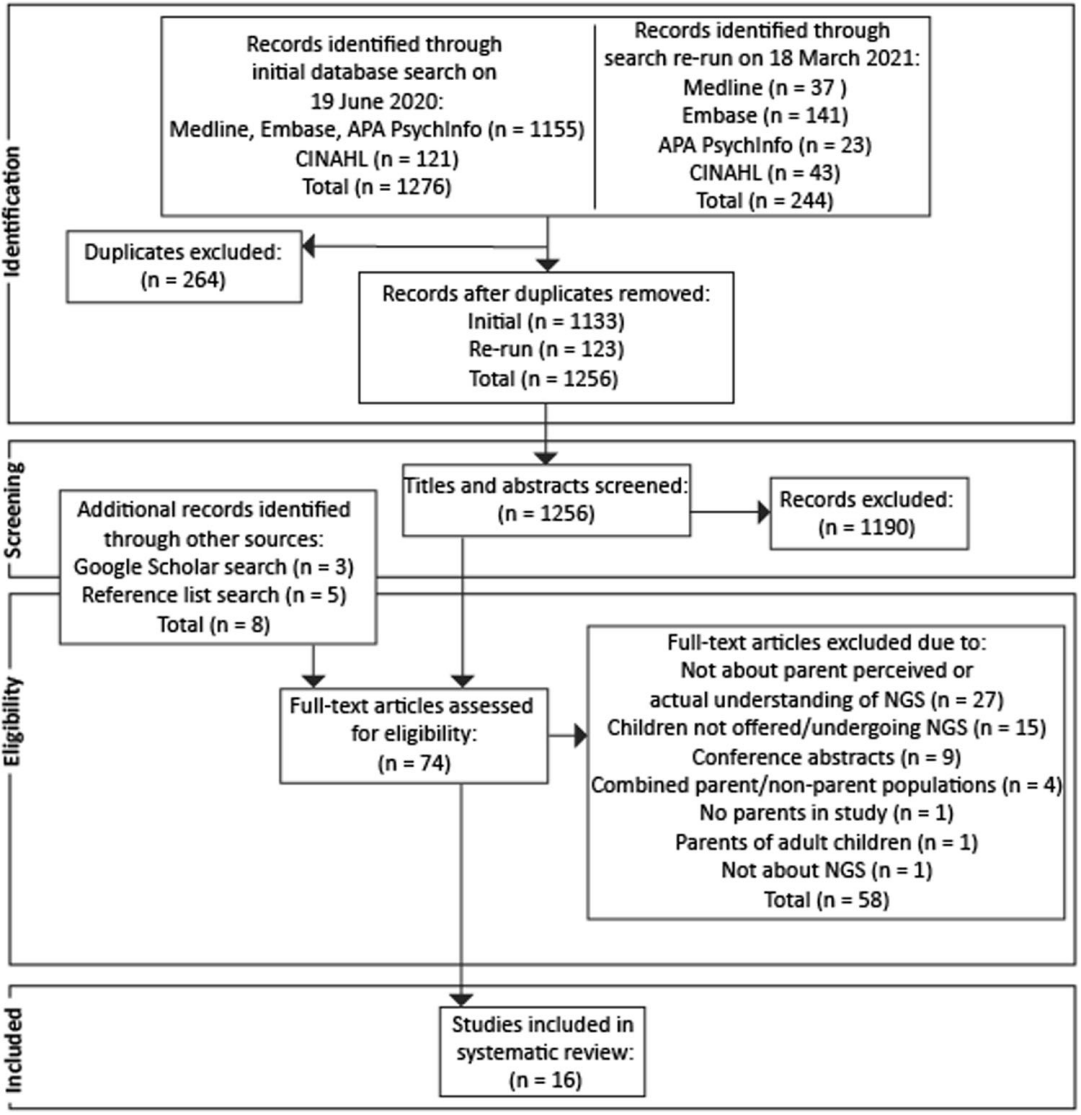
Systematic Review PRISMA flow diagram. This diagram details the process of identifying records following database searches, and screening of abstracts and full-texts for eligibility.

Two authors screened the titles and abstracts of the 1256 unique articles identified (Cohen’s k = 0.76, “almost perfect” 98% initial agreement), and full texts of remaining articles (Cohen’s k = 0.36, “fair” 65% initial agreement), and resolved discrepancies through discussion. Sixteen studies ultimately met full eligibility criteria. Refer to Fig. 1 for the PRISMA flow diagram. All articles included were of high quality and did not warrant exclusion based on quality assessment (Supplementary Table 2).

### Article characteristics

Table 1 summarizes key aspects of the 16 eligible articles, of which 15 [9, 18–31] evaluated parents’ actual understanding of GS/ES, and six [9, 18, 19, 28, 29] evaluated perceived understanding. Studies employed diverse methodologies and measures for exploring parents’ understanding of GS/ES (Table 2). Studies were conducted at various points in the patient’s clinical trajectory (Table 1). Two eligible studies were from the same broader UK study [19, 23], however included different parent cohorts and methodologies. Two eligible studies [9, 26] reported on different outcomes collected from the sample parent cohort as part of the same study; we counted data from these articles once.

**Table 1:** Overview of included studies.

First author, ref.	Country	Recruitment context	Type of sequencing	Counseling or information provided to parents in addition to the consent form	Parents with prior experience with genetic/genomic testing (%)	Parents (*n*)	Child’s diagnosis	Age of children (if/as reported in article)	Stage understanding was assessed at in relation to the return of results (Pre-, post-, or longitudinal)
Anderson et al. [30]	Canada	SickKids Genome Clinic clinical research project, pediatric genetics clinic	Germline GS	Counseling with geneticist or genetic counselor	Prior genetic testing (72%)	23	Developmental delay and/or congenital anomalies, complex disorders	0–6+ years, all “children”	Pre
Berrios et al. [31]	USA	NSIGHT clinical trial, intensive care units at one pediatric hospital	Germline GS	Not reported	Not reported	23	Suspected genetic condition with no unifying molecular or clinical diagnosis	<4 months	Post
Cakici et al. [32]	USA	NSIGHT2 clinical trial, intensive care units at one pediatric hospital	Rapid ES or GS, or ultrarapid GS	Consent consultation with research nurse, genetic counseling available (access rate not reported)	Not reported	312 (Pre), 157 (Post)	Unspecified	1–121 days	Pre-Post
Chassagne et al. [18]	France	Two genetics clinics	Germline ES	Consultations with geneticist	Prior genetic testing (67%)	570	Undiagnosed developmental disorders	Median age 7 years	Pre-Post
Dheensa et al. [19]	UK	100,000 Genomes Project (100 kGP) research project, children’s hospital genetics/genomics clinic	Germline GS	Consultations and written information	% not specified, however genetic tests identified as integral to clinical journey	16	Rare genetic diseases	Described as young children	Pre
Gal et al. [20]	USA	One pediatric hospital	Germline GS	Not reported	Prior genetic testing (60%)	47	Critical cardiac disease	<21 years	Mixed (Pre and Post)
Jaitovich Groisman et al. [21]	Canada	“Personalized medicine in the treatment of epilepsy” study, two pediatric hospitals	Germline GS	Consent consultation	Not reported	32	Pharmaco-resistant epilepsy	5 months–<18 years	Pre
Johnson et al. [22]	USA	Genomes for Kids (G4K) clinical trial	Germline GS	Consent consultations with genetic counselor-trained study nurse, study brochure and information sheet	Not reported	121	Cancer	Described as “pediatric patients”	Pre [2×]
Lewis et al. [23]	UK	100,000 Genomes Project (100 kGP) research project, children’s hospital genomics clinic	Germline GS	Study description, researcher discussion, participant information sheet	Not reported	20	Rare diseases	10 months–18 years	Pre
Luksic et al. [24]	USA	Children’s hospital genetics/genomics clinic	Germline ES	Consultations with treating geneticist, invitation letter	Prior genetic testing (82%)	38	Developmental delay, autism, and seizures, and other conditions	Mean 7 years, range 2–18 years	Post
Malek et al. [25]	USA	BASIC3 research study, children’s hospital	Germline + somatic ES	Consent consultation	Not reported	64	Cancer	Median 5.6 years, range 0.2–17 years)	Longitudinal (Pre and Post)
Marron et al. [9]	USA	iCAT clinical trial, four pediatric clinical settings	Somatic GS	Consent consultations with oncologist	Prior genetic testing (27%); prior genetics coursework (51%); other exposure to genetics (13%)	33	Recurrent, refractory, or high-risk extracranial solid tumor	<2–17 years	Post
Marron et al. [26]	USA	“	“	“	“	“	“	0–17 years	“
McConkie-Rosell et al. [27]	USA	Duke Genome Sequencing Clinic, research project	Germline ES	Pre-test and post-test genetic counseling, access to treating team	Not reported	19	Cancer, or other diagnosis	Reported age of first concern with child 0–3 years, and at time of study 2.8–16 years in search of diagnosis	Post
Rini et al. [28]	USA	NCGENES clinical trial	Germline ES	Consultations with study geneticists and genetic counselors, brochures	Prior genetic testing (82%)	116	Neurodegenerative disorders, cardiovascular diseases, dysmorphology, cancer	Age not specified, “pediatric patients”	Longitudinal study (2× Pre and 1× Post)
Tolusso et al. [29]	USA	Children’s hospital genetics and non-genetics specialty clinics	Germline ES	Consultation with either genetic counselor or other healthcare provider, brochure	Not reported	53	Patients from genetics and non-genetics specialty clinics	Median 7 years (genetics clinic), median 4 years (specialty clinic)	Mixed (Pre and Post)

*GS* Genome sequencing, *ES* exome sequencing.

**Table 2:** Summary of included studies’ measures and main findings on parents’ perceived and actual understanding of GS/ES.

First author, ref.	Study design	Summary of measures used to assess understanding
Anderson et al. [30]	Qualitative	SSI (understanding of NGS, information needs, motivations and expectations of participating
Berrios et al. [31]	Mixed methods (retrospective)	Qualitative: questions about the study purpose, enrollment process, attitudes towards pediatric NGS and research, previous genetic testing experience, whether a genetic diagnosis from NGS was received and the impact of the diagnosis Quantitative: Actual understanding—applied subscale from a validated genetic knowledge measure; combined Actual/Perceived understanding scores from awareness subscale of the Genetic Literacy and Comprehension (GLAC) Instrument
Cakici et al. [32]	Quantitative	15 question survey at two timepoints (immediately after enrollment, and within 1 week of return of genomic results); questions assessed: adequacy of consent, perceived utility of GS results, decisional regret
Chassagne et al. [18]	Mixed methods	Qualitative: SSI after return of results (understanding, expectations and reactions) Quantitative: questionnaire given prior to return of results
Dheensa et al. [19]	Qualitative	SSI with questions about experiences and motivations, hopes and concerns, perspectives on NGS research and broader use of genomic data, expectation of a diagnosis and consultation with other family members
Gal et al. [20]	Qualitative	SSI assessed previous experiences with and understanding of genetic testing, perceptions of clinical GS, thoughts about clinical genome sequencing
Jaitovich Groisman et al. [21]	Mixed methods	Mixed methods questionnaire addressing experiences with and understanding of child’s health condition, parent(s)’ decision-making regarding NGS
Johnson et al. [22]	Quantitative	Author-developed genetic/genomic knowledge questionnaire
Lewis et al. [23]	Qualitative	Semi-structured interview probing understanding of the purpose of genome sequencing, likelihood of a positive result, how genomic data will be later used, motivations and concerns, consent to secondary findings; information needs and satisfaction with the consent procedures and materials
Luksic et al. [24]	Qualitative	Semi-structured interview with questions probing the impact of testing, sharing of results, emotional reactions to and understanding of results, and specific questions about experiences with access to clinical care; authors categorized understanding of the results as: accurate, accurate plus, accurate minus, or inaccurate
Malek et al. [25]	Qualitative	Semi-structured interviews exploring the expected and perceived benefits of clinical WES
Marron et al. [9]	Quantitative	From a broader 103-item questionnaire, a 4-item questionnaire used to assess understanding of NGS (adapted from the Quality of Informed Consent measure)
Marron et al. [26]	Quantitative	From a broader 103-item questionnaire, selected data reporting on: respondent characteristics, hopes and concerns about profiling, understanding of profiling, results of participation, and preferences for return of results
McConkie-Rosell et al. [27]	Qualitative	Semi-structured interview, questions explored parental expectations and understanding of NGS and returned results, use of the NGS data, communication of findings to health/educational professionals and family members, information needs
Rini et al. [28]	Mixed methods	Longitudinal study with questionnaire; Actual understanding—questionnaire with University of North Carolina Genomic Knowledge Scale (questions about genes, genetic effects on health, familial inheritance, and diagnostic exome sequencing); Perceived understanding—six items probing perceived understanding of NGS
Tolusso et al. [29]	Quantitative	Survey modeled off Quality of Informed Consent (QuIC) questionnaire; Actual understanding—questions about NGS, secondary findings (scope, description, benefits, risks, voluntary, refusal, alternative test, confidentiality, future use, and secondary findings); Perceived understanding—questions assessing perceived understanding of WES and SFs

*SSI *Semi-structured interview.

### Sample characteristics

A total of 1487 parents (range of 16–570 parents per study) participated in the included studies, representing the views of parents of children affected by a range of health conditions (Table 1). Five studies reported that a significant proportion (at least 65%) of parents had prior experience with genetic testing, genetics-related coursework, or other exposures (Table 1) [9, 18, 24, 28, 30]. Many studies reported that in addition to being given the consent form, parents underwent some form of consent consultation with a healthcare professional with some degree of training in genetic counseling (Table 1).

### Parent actual understanding of GS/ES

In Table 3, we provide a summary of concepts that parents understood well and less well, organized using the Ayuso et al. [4] framework described above.

**Table 3:** Summary of data about parents’ actual understanding of GS/ES, organized using Ayuso et al.’s list of elements of information for GS/ES informed consent processes.

Elements	Well understood	Less well understood
Scope	• Higher chance of finding causative genetic variant than other genetic tests [18, 23, 29]	• What makes NGS “comprehensive” [23] • Key differences between NGS and other genetic tests [18, 23, 24, 27]
Description	• Genomic concepts [22, 28, 31] • Clinical significance of returned results [24, 27]	• De novo vs. inherited variants [28] • Somatic vs. germline variants [22] • Implications of a not finding a causative genetic variant [24]
Benefits	• Clarified diagnosis and/or prognosis [18–21, 23, 27] • Potential clinical utility of results [19, 21, 23, 25, 30, 31] • Potential psychological benefit for parents [20, 21, 23, 25, 31]	Not reported
Risks	• Family members could also have the variant [23–25, 30] • No guarantee of a clinically actionable result [9, 19, 21, 23] • Negative psychological impact of results [23–25, 31]	Not reported
Alternatives	Not reported	Not reported
Confidentiality	• Risk of insurance-related challenges (including potential future legal changes to protections) after receiving secondary findings [13, 19, 20, 23, 29, 30]	Not reported
Future use	• NGS (in context) was done to advance research to help future patients [9, 18, 19, 21–23, 30, 31] • Which institutions could store and access NGS data [23]	Not reported
Secondary findings (SFs)	• Indicates an elevated risk of developing another health condition [29] • Low chance of finding secondary variant [23] • Scope for family surveillance or history confirmation [23, 29, 30]	• Scope/definition of what SFs could be found [23, 29] • What SFs will be reported [23] • Autonomy in deciding what results to receive [23]

### Actual understanding of the scope of GS/ES

Many parents in one qualitative study showed limited understanding of the difference between ES and other genetic tests their child had previously undergone [24]. Some parents recalled being told about the comprehensive nature of ES, and that samples would be taken from the patient and parents [24]. Another study using a validated quantitative actual understanding instrument found parents had good understanding that the comprehensiveness of ES can overcome limitations of other prior genetic tests, increase the likelihood of finding a causative variant, and may also find a variant of uncertain significance [29]. This parent cohort, however, understood less well the depth of ES analysis of DNA, that ES carries a higher chance of returning a secondary finding [29]. Three studies using semi-structured interviews reported that several parents expressed a view of GS/ES as a simple blood test and diagnostic tool [18, 23, 27]. However, many parents correctly described key characteristics of testing and that an additional sample may be needed for testing [23, 27]. Parents understood that it could take a long time for results to come back after sample collection [26].

### Actual understanding of the description of genomic concepts and returned results

#### Genomic principles

Three studies reported good understanding of concepts queried using their respective quantitative measures of common genetic/genomic concepts [22, 28, 31]. One study found strong actual understanding of items about applied genetic concepts related to inheritance and the relationship of genes to health [31]. These parents attained genetic literacy scores comparable to a group from the general population [31]. Another study found over half of the parents around the time of recruitment correctly answered at least 75% of questions assessing understanding of 11 common genetic/genomic concepts [22]. Parents most frequently understood that genes are made of DNA, that genetic risk relates to inheritance or predisposition to a given genetic disorder, that a child can inherit a disease-causing genetic variant from two otherwise healthy parents, and that genome sequencing can return information that could impact both the child and other family members [22]. Another study which asked 25 questions assessing understanding of genes and health, genetic variant inheritance and ES found that parents could identify variant inheritance patterns, and had strong understanding that genetic variants detected would not necessarily be associated with causing disease [28]. Parents in this study showed lower understanding that a genetic variant causing a health condition could arise spontaneously (de novo), and that some variants can have a disease-prevention effect [28]. In one study using interviews, parents described how the technical nature of GS and their limited understanding of genetics impacted their approach towards decision-making following the return of their child’s results [20].

#### Clinical significance of results

Two qualitative studies reported on parent understanding of the clinical significance of returned ES results [24, 27]. In both studies, parents could accurately describe the clinical significance of their child’s returned ES results [24, 27]. Many parents demonstrated a comprehensive understanding of their child’s results, and correctly identified the detected variant’s inheritance pattern, or the implications of no variant being detected [24]. Other parents misunderstood the fact that not finding a disease-causing genetic variant does not mean the child’s condition does not have a genetic cause, nor does it eliminate the possibility that siblings or future children could have the same condition [24]. The second study reported good parent understanding of the chance of siblings of the patient carrying the same genetic variant identified through testing [27]. Parents were frequently unable to name the gene the variant was found in [27].

### Actual understanding of the potential benefits of GS/ES

Across six studies of various methodology, parents demonstrated understanding of GS/ES’ potential to enable disease classification and indicate a potential prognosis [18–21, 23, 27]. Most parents understood that a GS/ES result could lead to a change in their child’s clinical care (including improved treatment selection, recommendation of further testing, disease surveillance, treatment decisions, or palliation if appropriate) [19, 21, 23, 25, 30, 31]. Five studies highlighted parent understanding that a child’s GS results could be of psychological benefit to parents [20, 21, 23, 25, 31]. Parents reported the potential for satisfaction of curiosity [23, 25], relief of guilt [25, 31], peace of mind [25], and preparation for the future [21, 25, 31].

### Actual understanding of the potential risks of GS/ES

Several studies noted that at least some parents understood that there was no guarantee that GS would uncover a clinically actionable genetic variant [9, 19, 21, 23]. Some parents understood that GS/ES results could reveal a poor prognosis or more serious diagnosis than presently held, or result in psychological distress [23–25, 31]. Parents across four studies generally understood that a positive result could reveal a risk to other family members, and that a genetic variant known to be disease-causing could warrant surveillance or testing for other family members [23–25, 30].

### Actual understanding of the alternative diagnostic methods

A substantial proportion of parents across many studies already had prior experience with genetics-based tests (Table 1) [18, 19, 24, 26, 27, 30]. In one study, some parents identified that GS was the last resort for diagnosis after having exhausted all other relevant and accessible options [23].

### Actual understanding of the privacy and data confidentiality

Parents across five studies showed varying degrees of understanding of the risk of life insurance-related concerns (such as discrimination and potential future legal changes to protections related to secondary findings) [19, 20, 23, 29, 30]. One of these studies, conducted in the US reported that several parents appeared unaware of protections provided by the US congressional Genetic Information Nondiscrimination Act (GINA) of 2008 [20]. Some of these parents, however, showed understanding of potential risks that extend beyond the protections of this act [20].

### Actual understanding of the future use and storage of GS/ES data

Eight studies conducted in a research context reported that parents understood that their child was enrolled in a study that aimed to improve clinical outcomes for future patients [9, 18, 19, 21–23, 30, 31]. One qualitative UK study reported that parents understood that access to their child’s GS data could be granted to various commercial, pharmaceutical and research institutions, and stored in a national database [23]. Parents in this study demonstrated awareness of issues related to data security and privacy, some identifying that data would be protected through de-identification [23]. One study reported that some parents raised concerns about potentially participating in research with applications to which they were morally opposed, demonstrating an awareness of nuanced ethical issues associated with genomic databases [19].

### Actual understanding of the possibility of secondary findings

Three studies explored parents’ understanding of clinically significant variants unrelated to the primary indication however were intentionally searched for, and which may be reported to families [23, 29, 30]. In one US study of 53 parents, over half of the parents correctly answered at least seven out of nine questions probing actual understanding of secondary findings [29]. Parents in this study had strong actual understanding of the possibility of finding a genetic variant associated with an elevated risk of developing an additional health condition [29]. In another study, some parents specifically identified that the likelihood of finding a secondary finding was low [23]. Many parents across three studies understood that knowing about a secondary finding could help them to prepare for the onset of the associated health condition [23], give family members the option to test for the same variant [29, 30], or confirm family history of a known familial disease [23]. Two studies reported that parents understood that there could be a psychological or emotional impact of receiving a secondary finding [23, 30].

Two studies identified specific concepts related to secondary findings that were less well understood [23, 29]. Over half of the parents in one quantitative study incorrectly believed that secondary findings could be related to personal traits such as height and hair color, in addition to predisposition to other health conditions [29]. The second study similarly found through interviews that parents were unsure about the scope of health conditions that could be associated with secondary findings [23]. Many of these parents were unsure about whether they would be informed only of ‘clinically actionable’ secondary findings [23].

### Parents’ perceived understanding of GS/ES

Five studies explored parents’ perceived understanding of GS/ES, with mixed results (Table 2) [9, 18, 19, 28, 29]. One study which assessed parents’ perceived understanding of 17 concepts related to GS/ES found that each concept was understood by parents at least “Mostly” [29]. Parents more confidently perceived they understood: that undergoing ES was voluntary, which individuals would be tested, the benefits of ES results, and that receiving secondary findings would be optional [29]. Parents less confidently understood concepts concerning possible discrimination based on ES results, and implications of secondary findings [29].

One study reported that over 60% of parents felt they understood the consent conversations they had with their doctor about the study and the testing it involved “Well” or “Extremely well” [9]. Many parents enrolled in another study self-reported difficulty in understanding aspects of the GS project, but did not feel it was necessary to understand all information provided due to their trust in referring healthcare professionals [19]. In one study, the majority of parents indicated in surveys both at enrollment and after receiving their child’s results that they received adequate information to decide whether to enroll and whether to opt in to receive findings unrelated to their child’s health condition [32]. Most parents reported understanding the results returned, however there were significantly higher rates of understanding of negative results (no variant reported) as compared with positive results (variant reported) [32].

One US study of parents of children with diverse undiagnosed conditions found a positive correlation between scores of baseline actual understanding of concepts thought to be critical for deciding to undergo ES, and perceived understanding of similar informational concepts after the return of results [28]. This study also found higher levels of positive change in genomic understanding to be marginally associated with perceived understanding [28]. Another study reported that 10% of its parent cohort had a “good” or “very good” “knowledge of genetics”; however, it is unclear whether this finding was obtained using a measure of perceived (rather than actual) understanding [18].

### Factors associated with parent understanding of GS/ES

Five studies reported on sociodemographic factors found to be associated with parents’ (actual or perceived) understanding of GS/ES [9, 22, 24, 28, 29]. Some studies found stronger actual understanding to be associated with: higher levels of education [9, 24], higher genetic understanding [9], higher health literacy [28], higher income [28], proficiency in English [28], being of non-Hispanic white ethnicity [28], and higher acculturation to the dominant culture in the study context [24]. One study, however, found no significant association between actual understanding and ethnicity, age, sex, likelihood of child’s cure, receipt of a treatment recommendation, or perceived understanding of study information [9]. One study reported that parents recruited from a genetics clinic had higher levels of perceived understanding than and comparable levels of actual understanding to parents recruited from other specialty clinics [29].

Two US studies found that introduction of a genetic counseling consultation was associated with significant overall increases in actual understanding of genetic/genomic concepts [22, 28]. Consultations in both studies addressed concepts in study materials and parents’ questionnaire responses. One of these studies (which involved two consultations with a study nurse prior to each of two understanding assessments) found one third of parents had a persistent misunderstanding of the distinction between somatic (tumor) and germline (non-tumor) variants [22]. The second study had both an additional consultation and understanding assessment timepoint, and found negligible increases in actual understanding from the second to third questionnaires [28].

## Discussion

With the increasing implementation of GS/ES as a diagnostic test in pediatric care, it is important that we evaluate how well parents of children offered GS/ES understand related concepts. This systematic review synthesized findings from 16 studies which explored parents’ actual and perceived understanding of GS/ES, and factors associated with understanding. Studies either explicitly assessed parents’ responses to questions probing understanding, or implicitly assessed understanding through open-ended questions (e.g., asking about benefits or concerns regarding GS/ES).

Overall, parents from studies included in this systematic review demonstrated some understanding of the various potential current and future applications of their child’s GS/ES data [9, 18–23, 25, 27, 30, 31]. This, however, does not imply depth nor accuracy of understanding, especially of the likelihood that their child will derive any direct clinical benefit from undergoing GS/ES. We also found many parents have a realistic understanding of the likelihood of GS/ES returning a clinically actionable result [9, 19, 21, 23]. However, the broader literature indicates that realistic understanding of context-specific limitations of comprehensive genetic testing is not universal [5]. Parents have been found to overestimate the likelihood of a novel genetics-based technology yielding a favorable clinical outcome, especially after other genetics-based options were unsuccessful [5]. Adult patients can also have unrealistic expectations of achieving a favorable outcome following GS/ES [33]. Adult patients [34], like parents in this review [26, 35], more readily understand concepts related to heredity than those related to insurance discrimination and the nature of secondary findings. Clinicians have also recognized that misunderstanding of GS/ES’ applications by adult patients and parents of pediatric patients alike constitutes a primary challenge to facilitating informed consent processes [3, 6, 34]. Areas of weaker understanding may be addressed through consultations led by healthcare professionals who have undertaken appropriate training, as evidenced by two studies included in this review [22, 28], additional literature exploring efficacy of genetic counseling interventions [36], and physicians’ self-reported confidence in understanding genomic sequencing [37].

It is unclear whether parents’ understanding of the potential utility of GS/ES is matched with a realistic appreciation of GS/ES’ present applications in clinical contexts. The literature acknowledges that the treatment of risk versus benefit discussions in clinical consultations between parents and their child’s healthcare professionals can influence parents’ understanding of these concepts [25]. The perceived focus on benefits in the included studies could be due to a conflation between parents’ hope for fulfillment of a desired outcome, with expectations of what the outcome is most likely to be [38]. Also important to consider is the emotional intensity of the situation in which GS/ES could be conducted, compounded by potentially needing to make difficult decisions in time-pressured situations regarding procedures and future care plans [8, 20]. Further, higher levels of education were found to be strongly associated with actual understanding of GS/ES’ limitations [34]. This study did not find parents’ level of education to be associated with understanding of the potential benefits [34].

Our review has synthesized evidence that parents’ perceived understanding of GS/ES information is highly variable. The literature suggests that perceived understanding can be highly context-dependent, and assumptions cannot be made about someone’s perceived understanding from their actual understanding scores alone [39]. However, evidence shows that adult patients enrolled in clinical trials may be inclined to have lower perceived understanding of risks associated with trial participation [40]. A study of adult cancer patients suggests that delaying signing of the consent form until after the initial consultation to discuss GS/ES can increase perceived understanding [41]. This study also found that higher perceived understanding was associated with higher satisfaction with decisions made regarding clinical care [41]. Perceived understanding is a less-explored outcome in the literature, and further research into parents’ perceived understanding of GS/ES in pediatric contexts is warranted.

Variable parent understanding of GS/ES highlights the need to better support healthcare professionals to conduct effective consultations with parents offered GS/ES, gauge parents’ understanding and motivations for participating, and provide ongoing support as deemed appropriate [6, 19]. Studies included in this review found higher levels of actual understanding to be associated with factors such as parents’ level of education, understanding of genetics, health literacy, and cultural and linguistic background [9, 24, 28]. The literature broadly appears to recommend use of consent processes that accommodate the diverse needs of families [42], and consider that complex information is more difficult to retain for individuals experiencing a heightened state of emotion or distress [8]. Management of expectations for receiving a diagnosis should be considered within the context of diagnostic yield discrepancies across disease groups [43].

### Limitations

Our systematic review contributes to the literature responding to the increasing implementation of GS/ES in pediatrics, however should be considered in light of several limitations. Our article inclusion criteria restricted eligible studies to those available in English, and to inclusion of parents of a pediatric patient either offered or undergoing GS/ES. Defining and classifying what constitutes “understanding” of GS/ES was a challenge due to the many complexities surrounding GS/ES. The heterogeneity of included studies’ methodologies used (both qualitative and quantitative), outcomes assessed, and study contexts (research, clinical or hybrid, disease groups, and diversity of informed consent consultations) limited our ability to assess the depth, prevalence, and accuracy of parents’ understanding across the respective parent cohorts. Included studies were conducted at different stages in the patient’s diagnostic journey, therefore precluding direct comparison of parent understanding at different timepoints. Perceived understanding data from included studies were more limited than actual understanding data, therefore could not be organized using the same framework. Additionally, due to both methodological heterogeneity and limited availability of recruitment setting information, we were not able to account for differences between parent samples, such as the nature and level of counseling provided, access to informational resources, or various sociodemographic differences.

### Future research

Future studies should consider implementing a comprehensive assessment of both parents’ perceived and actual understanding of GS/ES concepts. While it is difficult to reliably assess actual understanding across studies, even more so across different populations and diagnoses, there is value in using validated measures of understanding wherever possible and identifying improved tools for querying understanding. Future studies could simultaneously investigate the acceptability of interventions such as genetic counselor appointments geared towards improving understanding. At present, research is underway that explores additional outcomes to assess alongside parent understanding, such as measures of therapeutic misconception, hopes and expectations, distress, regret, and health literacy [44]. Future research is warranted into associations between individuals’ satisfaction with the amount and nature of information received, perceived understanding, and psychosocial factors such as distress and anxiety.

## Conclusion

Parents readily appreciate the potential benefits of their child undergoing GS/ES. However, there is scope for improving understanding of potential associated risks especially of secondary findings, and of use of data beyond their child’s clinical care. We summarize evidence of variability in parents’ perceived understanding of GS/ES, with many parents reporting difficulties understanding information provided about GS/ES. Our findings may assist clinicians to better support parents to understand GS/ES and the implications for their child.

## Supplementary information

Supplementary
