# Supplementary material for: Parents’ understanding of genome and exome sequencing for pediatric health conditions: a systematic review
Source: Eur J Hum Genet. 2022 Aug 23;30(11):1216–25. doi: 10.1038/s41431-022-01170-2 (PMC9626631; doi:10.1038/s41431-022-01170-2)
Supplement: Supplementary file 1 — Supplementary [file 41431_2022_1170_MOESM1_ESM.docx]

**SUPPLEMENTARY**

Supplementary Methods

**Systematic Review Search Strategy**

An initial search of the literature was conducted on 19 June 2020, and a re-run of the search conducted on 18 March 2021. Due to institutional changes in response to the COVID-19 situation, access to databases was changed. Both searches are equivalent, only facilitated by a different means of access.

**Initial search:**

*Using the Wolters Kluwer Ovid Platform*

Databases: Ovid MEDLINE®, Embase, APA PsycInfo

1. (child* or pediatric* or paediatric* or adolescent* or young adult* or AYA*).mp.

2. (sequenc* or next generation sequencing or whole exome or whole-exome or whole genome or WGS or WES).mp.

3. (parent* or caregiver* or carer* or guardian*).mp.

4. (understand* or perception* or comprehension or expectation* or attitude* or perspective*).mp.

5. 1 and 2 and 3 and 4

6. remove duplicates from 5

7. limit 6 to english language

8. limit 7 to yr="2008 -Current"

*CINAHL*

AB ( child* or pediatric* or paediatric* or adolescent* or young adult* or AYA* ) AND AB ( sequenc* or next generation sequencing or whole exome or whole-exome or whole genome or WGS or WES ) AND AB ( parent* or caregiver* or carer* or guardian* ) AND AB ( understand* or perception* or comprehension or expectation* or attitude* or perspective* )

Limiters – Date Published: 20080101-20201231

Expanders – Apply equivalent subjects

Narrow by Language: English

Search modes: Boolean/Phrase

*Google Scholar*

(note that Google Scholar is limited in the number of key words permissible in a search)

(child or pediatric) AND (sequencing or exome or genome) AND (parent or caregiver) AND (understanding or perception)

- first 200 records (20 pages) of search results screened

(sequencing or exome or genome) AND (parent or caregiver) AND (understanding or perception)

- first 200 records (20 pages) of search results screened

**Search re-run:**

*Embase using the OVID platform*

1. (child* or pediatric* or paediatric* or adolescent* or young adult* or AYA*).mp.

2. (sequenc* or next generation sequencing or whole exome or whole-exome or whole genome or WGS or WES).mp.

3. (parent* or caregiver* or carer* or guardian*).mp.

4. (understand* or perception* or comprehension or expectation* or attitude* or perspective*).mp.

5. 1 and 2 and 3 and 4

6. remove duplicates from 5

7. limit 6 to english language

8. limit 7 to yr="2020 -Current"

*APA PsychInfo using the ProQuest platform*

(child* OR pediatric* OR paediatric* OR adolescent* OR young adult* OR AYA*) AND (sequenc* OR "next generation sequencing" OR "whole exome" OR whole-exome OR "whole genome" OR WGS OR WES) AND (parent* OR caregiver* OR carer* OR guardian*) AND (understand* OR perception* OR comprehension OR expectation* OR attitude* OR perspective*) AND la.exact("English") AND PEER(yes)

Limited to publishing date: After 01 January 2020

Language: English

*CINAHL using the EBSCOhost platform*

AB ( child* or pediatric* or paediatric* or adolescent* or young adult* or AYA* ) AND AB ( sequenc* or next generation sequencing or whole exome or whole-exome or whole genome or WGS or WES ) AND AB ( parent* or caregiver* or carer* or guardian* ) AND AB ( understand* or perception* or comprehension or expectation* or attitude* or perspective* )

Limited to date published: 20200101-20211231

Expanders: Apply equivalent subjects

Language: English

*MEDLINE via the PubMed platform*

(((child*[tw] OR pediatric*[tw] OR paediatric*[tw] OR adolescent*[tw] OR young adult*[tw] OR AYA[tw] OR AYAs[tw]) AND (sequenc*[tw] OR "next generation sequencing"[tw] OR "whole exome"[tw] OR "whole-exome"[tw] OR "whole genome"[tw] OR "WGS"[tw] OR "WES"[tw])) AND (parent*[tw] OR caregiver*[tw] OR carer*[tw] OR guardian*[tw])) AND (understand*[tw] OR perception*[tw] OR "comprehension"[tw] OR expectation*[tw] OR attitude*[tw] OR perspective*[tw])

Limited to publishing date: 2020-2021

Language: English

Publications from MEDLINE-indexed journal

Supplementary Table 1: **Further details on Ayuso *et. al.*’s framework of elements of information to be covered in GS informed consent processes, and details of what these elements may contain**

**This includes discover of a variant which could reveal an unknown health risk to other family members*

| **Elements** | **Explanation of element** |
| --- | --- |
| Scope | aspects unique to comprehensive NGS as distinct from hypothesis-based genetic tests such as single gene and gene panel testing |
| Description | genomic testing principles, clinical significance of NGS results |
| Benefits | potential benefits to patient or family (clinical and psychosocial) |
| Risks | potential risks to patient or family (clinical and psychosocial)* |
| Voluntary & Refusal | voluntary nature of the test, possibility to refuse or opt-out |
| Alternatives | description of alternative diagnostic methods |
| Confidentiality | confidentiality of results, data inclusion in patient medical records, who will have access to the data |
| Future Use | storage of data, data usage for research purposes |
| Secondary Findings | management of secondary findings that appear in NGS data analysis, what can be reported, right not to know |

Supplementary Table 2: **Quality assessment of included studies using Mixed Methods Appraisal Tool (MMAT)**

| **First author, Year** | 1. Qualitative | | | | | 2. Quantitative randomized controlled trials | | | | | 3. Quantitative nonrandomized | | | | | 4. Quantitative descriptive | | | | | 5. Mixed methods | | | | |
| --- | --- | --- | --- | --- | --- | --- | --- | --- | --- | --- | --- | --- | --- | --- | --- | --- | --- | --- | --- | --- | --- | --- | --- | --- | --- |
|  | .1 | .2 | .3 | .4 | .5 | .1 | .2 | .3 | .4 | .5 | .1 | .2 | .3 | .4 | .5 | .1 | .2 | .3 | .4 | .5 | .1 | .2 | .3 | .4 | .5 |
| Anderson, 2017 | Y | Y | Y | Y | Y |  |  |  |  |  |  |  |  |  |  |  |  |  |  |  |  |  |  |  |  |
| Berrios, 2020 | Y | Y | Y | Y | Y |  |  |  |  |  |  |  |  |  |  | Y | Y | Y | Y | Y | Y | Y | Y | Y | Y |
| Cakici, 2020 |  |  |  |  |  |  |  |  |  |  | Y | Y | Y | Y | Y |  |  |  |  |  |  |  |  |  |  |
| Chassagne, 2019 | Y | Y | Y | Y | Y |  |  |  |  |  |  |  |  |  |  | Y | Y | Y | Y | Y | Y | Y | Y | Y | Y |
| Dheensa, 2019 | Y | Y | Y | Y | Y |  |  |  |  |  |  |  |  |  |  |  |  |  |  |  |  |  |  |  |  |
| Gal, 2021 | Y | Y | Y | Y | Y |  |  |  |  |  |  |  |  |  |  |  |  |  |  |  |  |  |  |  |  |
| Jaitovich Groisman, 2019 | Y | Y | Y | Y | Y |  |  |  |  |  |  |  |  |  |  | Y | Y | Y | Y | Y | Y | Y | Y | Y | Y |
| Johnson, 2019 |  |  |  |  |  |  |  |  |  |  | Y | Y | Y | Y | Y |  |  |  |  |  |  |  |  |  |  |
| Lewis, 2020 | Y | Y | Y | Y | Y |  |  |  |  |  |  |  |  |  |  |  |  |  |  |  |  |  |  |  |  |
| Luksic, 2020 | Y | Y | Y | Y | Y |  |  |  |  |  |  |  |  |  |  |  |  |  |  |  |  |  |  |  |  |
| Malek, 2017 | Y | Y | Y | Y | Y |  |  |  |  |  |  |  |  |  |  |  |  |  |  |  |  |  |  |  |  |
| Marron, 2019 |  |  |  |  |  |  |  |  |  |  |  |  |  |  |  | Y | Y | Y | Y | Y |  |  |  |  |  |
| Marron, 2016 |  |  |  |  |  |  |  |  |  |  |  |  |  |  |  | Y | Y | Y | Y | Y |  |  |  |  |  |
| McConkie-Rosell, 2016 | Y | Y | Y | Y | Y |  |  |  |  |  |  |  |  |  |  |  |  |  |  |  |  |  |  |  |  |
| Rini, 2020 |  |  |  |  |  |  |  |  |  |  |  |  |  |  |  | Y | Y | Y | Y | Y |  |  |  |  |  |
| Tolusso, 2017 |  |  |  |  |  |  |  |  |  |  |  |  |  |  |  | Y | Y | Y | C | Y |  |  |  |  |  |
